# Biosafety and Antibacterial Ability of Graphene and Graphene Oxide In Vitro and In Vivo

**DOI:** 10.1186/s11671-017-2317-0

**Published:** 2017-10-12

**Authors:** Long Pang, Chunqiu Dai, Long Bi, Zhongshang Guo, Junjun Fan

**Affiliations:** 1 The 3rd Orthopedic Department of General Hospital, Ningxia Medical University, Ningxia, 750004 China; 2 Department of Orthopaedics, Xijing Hospital, Fourth Military Medical University, No. 15 West Changle road, Xi’an, 710032 China; 3 Hanzhong Central Hospital, No. 22 Kangfu road, Hanzhong, 723099 China; 4 The Fifth Camp of the First Cadet Brigade, Fourth Military Medical University, Xi’an, 710032 People’s Republic of China

**Keywords:** Graphene, Graphene oxide, Nanoparticles, Bone marrow mesenchymal stem cells, Biocompatibility, Antibacterial ability

## Abstract

In recent years, graphene (G) and graphene oxide (GO) nanoparticles have begun to be applied in surgical implant surface modification. However, biosafety and antibacterial ability of G and GO are still unclear. In this study, the biosafety of G and GO in vitro was evaluated by co-culture with bone marrow mesenchymal stem cells (BMSCs) and biosafety in vivo was observed by implanting materials into mice muscle tissue. Biosafety results showed that 10 μg/ml was the safety critical concentration for G and GO. When the concentration was more than 10 μg/ml, the cytotoxicity of G and GO showed a dose-dependent manner.

Antibacterial results showed that G presented the antibacterial ability with the concentration equal to and more than 100 μg/ml; GO presented the antibacterial ability with the concentration equal to and more than 50 μg/ml. The antibacterial effect of G and GO were in a dose-dependent manner in vitro.

The GO or G concentration between 50 and 100 μg/ml may be the better range to keep the balance of cytotoxicity and antibacterial ability. Our study reveals that G and GO have potential to be used in clinic with good biosafety and antibacterial properties in a certain concentration range.

## Background

In recent years, surgical implants are widely used to treat bone fracture and other diseases, but the implant needs both good biosafety and antibacterial properties to avoid rejection and infection. In fact, orthopedic treatment of infective bone defect is still a major problem. On the aspect of bacteria, *Staphylococcus aureus* is the most common pathogen in orthopedics and orthopedic implant [1]. Due to bone defect and infection [2], the treatment is difficult and patients need a long time to be healed. If the wound does not heal, the last treatment is limb amputation [3, 4].

Good treatment of infective bone defect should satisfy both infection control and reconstruction of bone defect repair request simultaneously. With the development of bone tissue engineering, an increasing number of biomaterial applications are used in the field of orthopedic treatment. The cure rate of infection in the bones can be, therefore, greatly improved. These materials mainly include heterogeneous bone [5], bio-ceramics [6] (such as hydroxyapatite [7] and calcium phosphate [8]), polymers [9, 10], protein materials (such as collagen fibers [11]), and so on. Alongside these materials, Beatriz Pelaz et al. revealed the importance and promising prospect of nanotechnology in implants [12]; among these nanoparticles, graphene and its derivatives are other novel materials to meet the requirements for bone repair.

Graphene is two-dimensional, with a single or few layers of carbon atoms in a honeycomb structure [13–15]. It is widely used in composite materials [16, 17], sensors [18, 19], energy [16, 20], and other fields due to its excellent physical properties. Graphene oxide is a surface-functionalized graphene material which is in a layer of carbon atoms connected with two-dimensional infinite extension of the base surface-active groups containing oxygen and its graphene oxide form [21]. Graphene (G) and its derivatives have caused great concern in the biomedical field due to its unique two-dimensional structure, as well as specific physical and chemical properties [22]. Functionalized graphene and its derivatives have many functions such as drug loading [23], antibacterial [24], bioimaging [25, 26], and cancer therapy [27].

On the aspect of antimicrobial capacity, Li et al. revealed that G antimicrobial mechanism is mainly caused by charge transfer [28] and bacterial migration. Bacteria transferred to the surface of sharp nanosheets, which lacerates bacteria by the sharp edges [29]. Moreover, Tu et al. also demonstrated another potential antimicrobial mechanism that G can penetrate into the cells, leading into the extraction of large amounts of phospholipids from the cell membranes [30]. Thus, G and graphene oxide (GO) have bioactivity and antimicrobial capacity, which meets the requirements to be qualified as bone repair materials.

However, with large-scale production and application, graphene’s biosafety issues are particularly important. Workers may suffer from the exposure to nanoparticles (NPs) through multiple mediums including inhalation, cutaneous contact, and gastroenteric pathways. Andrea Prodi et al. suggested a stepwise approach to assess NP exposure for further protection [31]. Except for assessment, biosafety and biocompatibility are other research key points. Kan Wang et al. demonstrated the biocompatibility of GO, which exhibits toxicity to human fibroblast cells when the dose is less than 20 μg/ml but exhibits obvious cytotoxicity when dose is more than 50 μg/ml, with significantly decreasing cell adhesion [32]. At present, a more consistent view confirms that G and GO have a toxic effect on bacteria but at odds with toxic effect on cells [33–36]. G and GO’s function and toxicity still need more specific study. Beatriz Pelaz et al. raised a question, “how to reduce risks and to increase benefits are vital for the development of safe and effective nanomedicines,” which reminds and urges the study towards combination of G and GO’s potential risks and antibacterial ability in vivo and in vitro [12].

Bone marrow mesenchymal stem cells (BMSCs) are multipotent adult stem cells. They have become an important cell source for repairing bone defect in tissue engineering [37, 38]. Moreover, the interaction between graphene and derivatives and stem cell still lacks of research [39, 40].

Therefore, this study researched the effect of G and GO on BMSCs in vitro mice muscle tissues, *Staphylococcus aureus*, aiming to investigate cytotoxicity and antibacterial ability of G and GO in vivo and in vitro and to promote the research of carbon nanomaterial nanomedicine and nanotoxicity.

## Results

### G and GO Cytotoxicity

#### G and GO Cytotoxicity In Vitro

**Fig. 1:**
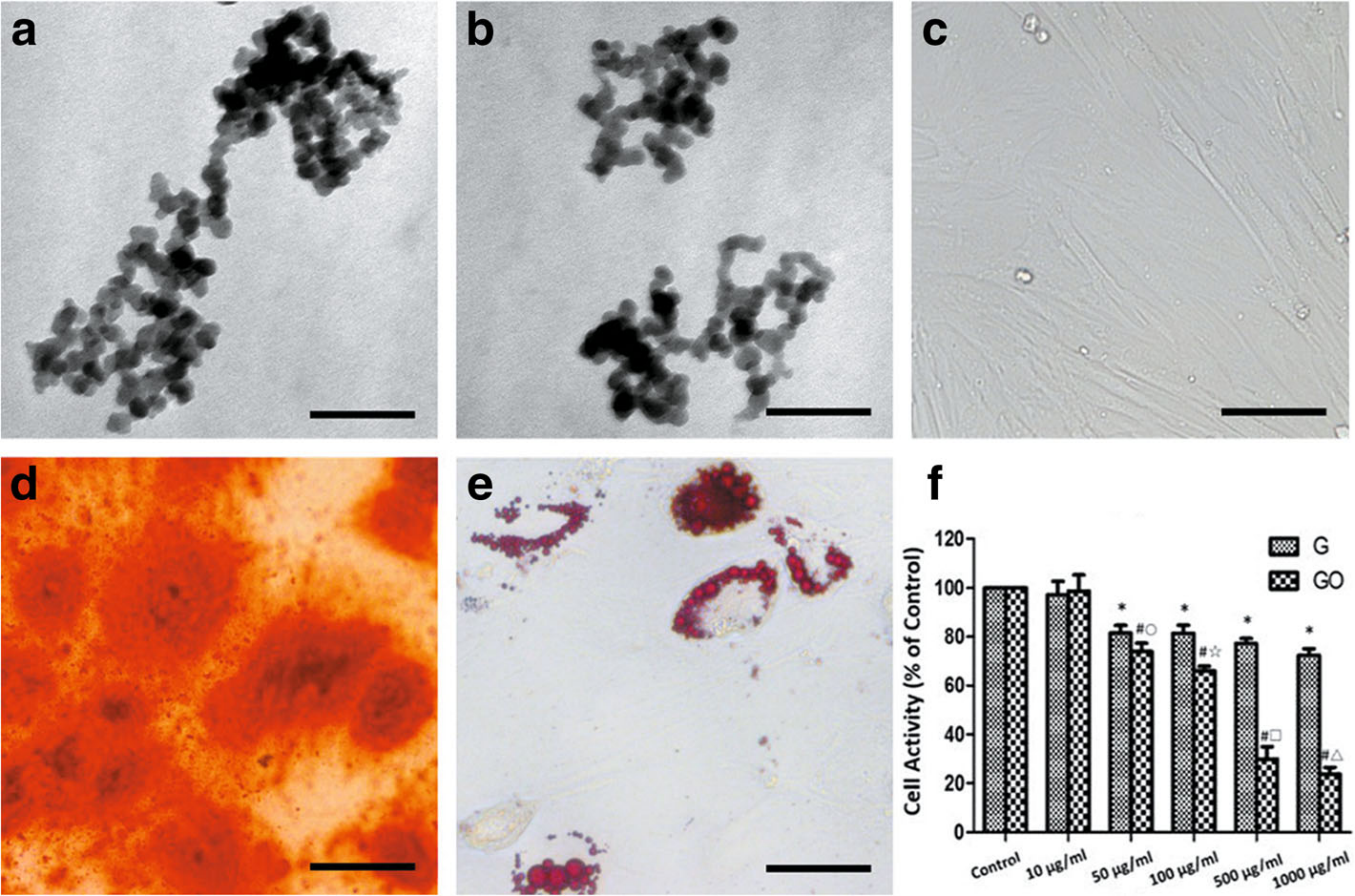
G and GO cytotoxicity (**a**, **b).** TEM images of G (**a**) and GO (**b**) showed the nano-network formed. **c** Cytomorphology of BMSCs. **d** Alizarin red for calcium deposition. **e** Oil red O for lipid. **f** Cell activity after G and GO treatment, *P < 0.01 with control group, ^#^ *P* < 0.01 with control group, ^○^ *P* < 0.05 with G 50 μg/ml group, ^☆, □, △^ *P* < 0.01 with the same concentration of G group. *r*2 (G) = 0.843, *r*2 (GO) = 0.939. Scale bars **a**, **b** 200 nm, **c**, **d** 100 μm, **e** 50 μm. *r*, correlation coefficient

Under electron microscope, G or GO nanoparticles showed irregular shape with the size of 30.41 ± 5.59 nm, and particle agglomeration could be found (Fig. 1a, b). After 7 days’ culture, morphology of the cell was changed into spindle shape (Fig. 1c). Calcium nodules were formed after culture with osteogenic differentiation medium (Fig. 1d). Oil accumulation was formed after adipogenic differentiation (Fig. 1e).

When the concentration was higher than 10 μg/ml, G or GO inhibited the growth of BMSCs. The cytotoxicity was the highest in 1000 μg/ml group and showed the dose-dependent manner. When the concentration was higher than 10 μg/ml, cytotoxicity of GO group was higher than G group at the same concentration. The difference was more significant with the concentration increasing (Fig. 1f).

**Fig. 2:**
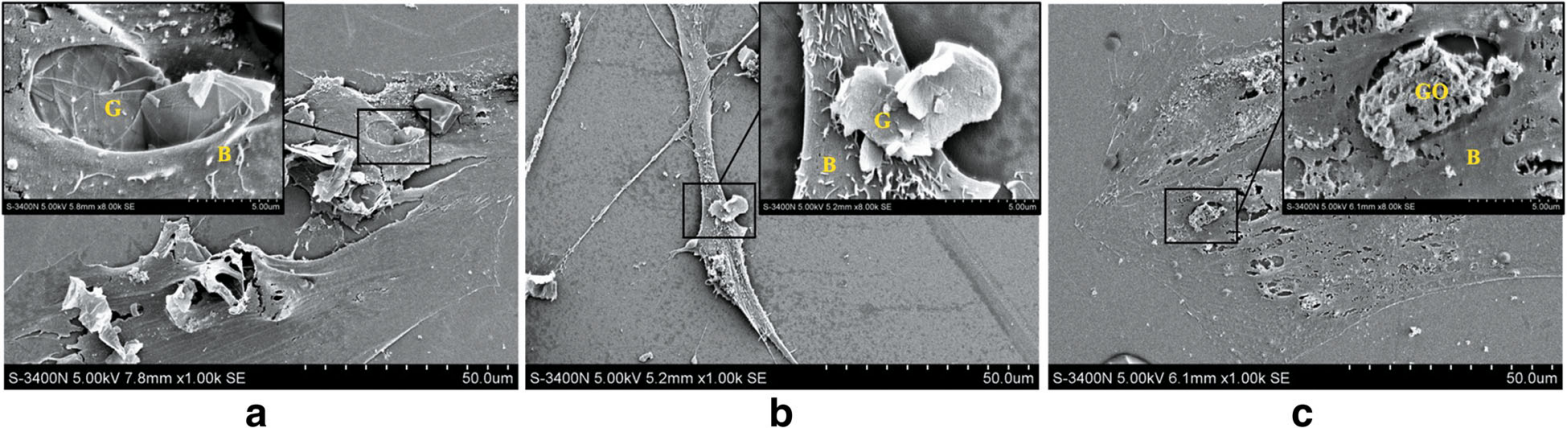
SEM images of co-culture of G, GO, and BMSCs. **a** G group, 10 μg/ml. The cells are in good condition. **b** G group, 50 μg/ml. Cell size decreases, surface secretion increases, and microvillus on the cell surface becomes long. **c** GO group, 50 μg/ml. BMSCs shrink and deform. G graphene, GO graphene oxide, B BMSCs

Under the observation of SEM, when the concentration of G or GO was 10 μg/ml, BMSCs were in good condition with good adhesion and shape. When the concentration of G was more than 50 μg/ml, cells were found to change including size decreasing, surface secretion increasing, and cell surface microvillus extension. When the concentration of GO was more than 50 μg/ml, BMSCs were found shrunken and deformed and most cells were dead. These results indicated that GO had higher cytotoxicity to BMSCs compared with G under the same concentration (Fig. 2).

**Fig. 3:**
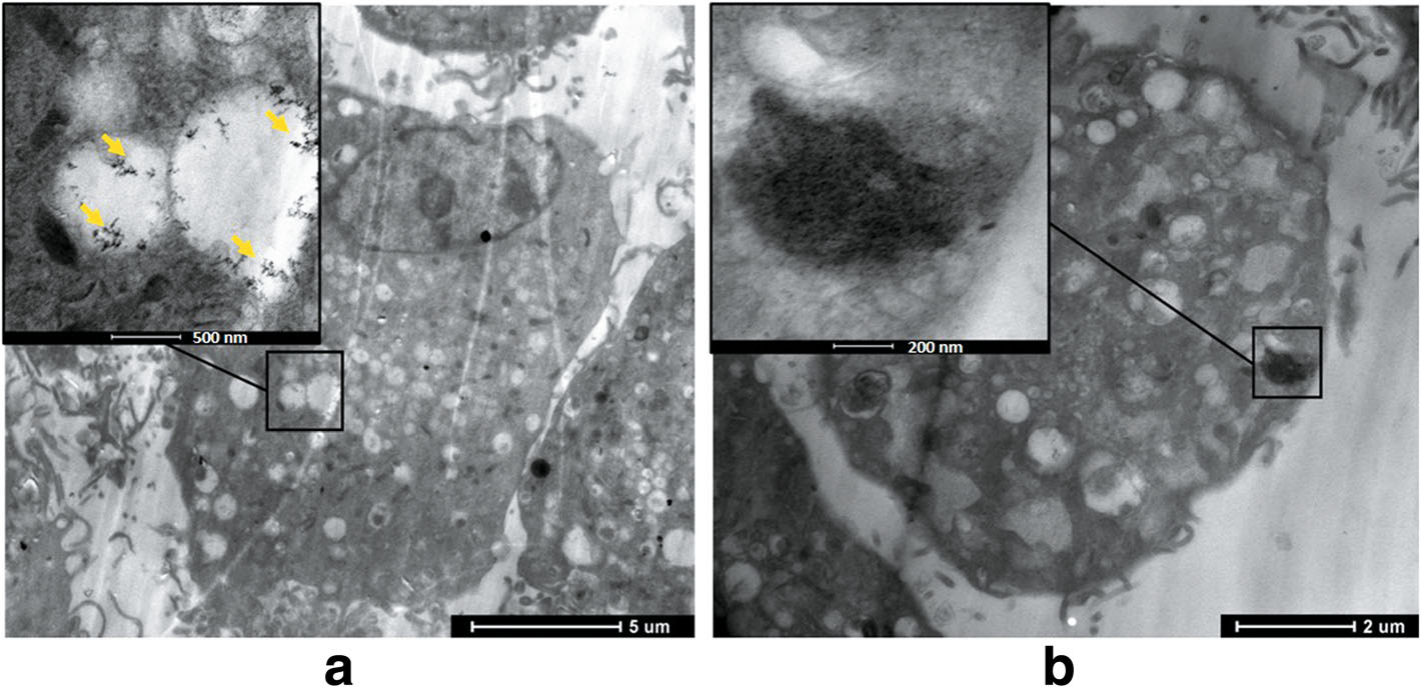
TEM images of co-culture of G, GO, and BMSCs. **a** G group. **b** GO group. Both G and GO can lead to cell structure disorder and microvillus mess on the cell surface; GO causes high cytotoxicity cellular microenvironment changes

Under the observation of TEM, we found G or GO could get into BMSCs and deposit on the cell internal. And when the concentration was more than 50 μg/ml, cellular microenvironment changes including cell structure disorder, and microvillus mess were found, indicating the higher cytotoxicity of GO compared with the G group (Fig. 3).

Based on the result of SEM and TEM observation, we found that 10 μg/ml was the safety critical concentration for G and GO. When the concentration of GO was more than 10 μg/ml, GO had higher cytotoxicity to BMSCs compared with G.

#### G and GO Cytotoxicity In Vivo

To analyze the G and GO cytotoxicity in vivo, we select skeletal tissue to represent and simulate local transplantation circumstance in orthopedics. The result of HE staining of skeletal tissue in the control group and G group presented the normal structure with muscle myofibrils parallel with vertical axis. In cross section, the myofibrillar section presented as the thin spots and nucleus was located at the edge of cells. The changes mentioned above could also be found in normal skeletal cells, indicating that G has little toxicity towards muscle tissues.

**Fig. 4:**
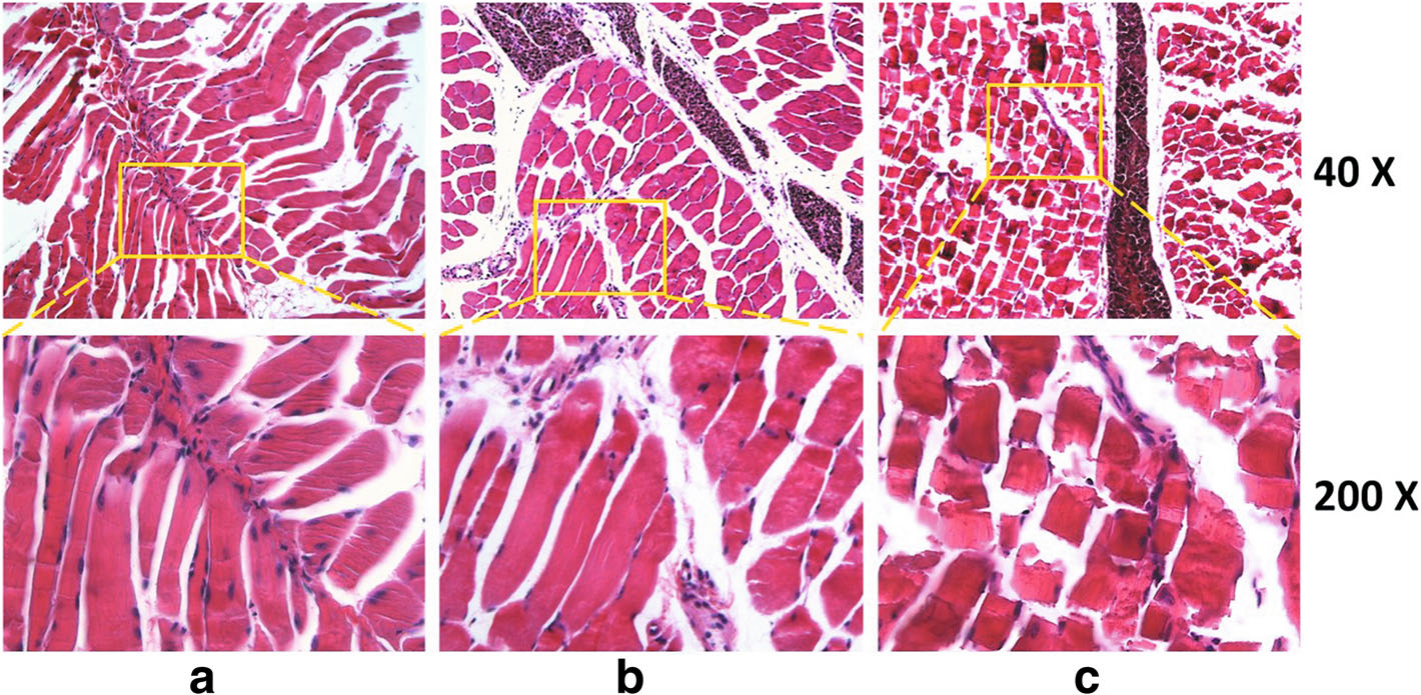
Tissue sections stained with HE staining. **a** Control represents uninjured tissue. **b** G group. Skeletal cells present as the straight strip. Muscle myofibrils are parallel along the long axis, transverse lines are clear, and cross section is irregular blocks. Myofibrillar section presents as the thin spots; nucleus is located at the edge. **c** GO group. Transverse lines of the muscle fibers in longitudinal section are fractured and not clear

On the contrary, in GO group, transverse lines of the muscle fibers in longitudinal section were fractured and not clear, revealing the muscle’s atrophy and necrosis. Thus, GO had higher toxicity to animals (Fig. 4).

### G and GO Antibacterial Properties

#### Antibacterial Ability In Vitro

**Fig. 5:**
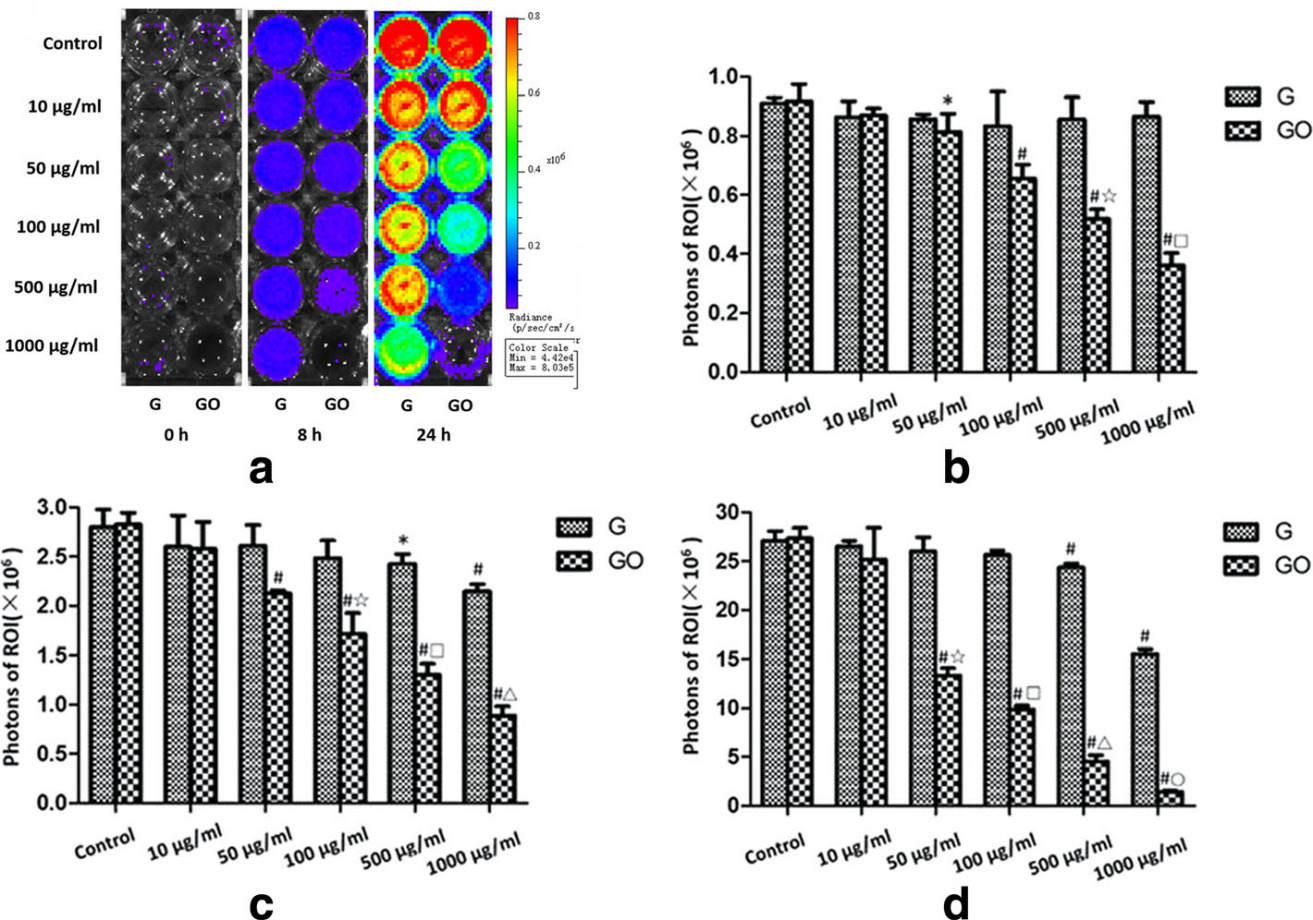
Intensity monitoring of bioluminescence of *S*. *aureus* in vitro. G and GO show a dose-dependent manner in antibacterial ability in vitro. **a** Bioluminescence of Xen-29 imaged in vitro after 0, 8, and 24 h of incubation at 37 °C, with variations in color representing light intensity (Bin M(8), FOV12, f1, 15 s). **b** PI = 0 h, *r*2 (GO-0 h) = 0.924. **c** PI = 8 h, *r*2 (G-8 h) = 0.584, *r*2 (GO-8 h) = 0.960. **d** PI = 24 h, *r*2 (G-24 h) = 0.616, *r*2 (GO-24 h) = 0.943.*P < 0.01 with control group, ^#^ *P* < 0.01 with control group, ^☆, □, △, ○^ *P* < 0.01 with the same concentration of G group. *r*, correlation coefficient

In the bacteriostasis experiment in vitro, the photon intensity of the ROI of G or GO showed a dose-dependent manner. And the photon intensity decreased in line with the concentration increasing. When compared with the G group at the same concentration, the GO group had lower photon intensity (Fig. 5).

At 0, 8, and 24 h, when the concentrations of G were 100, 500, and 1000 μg/ml, G showed the inhibition ability towards Xen-29 growth compared with the control group. However, the photon intensity in 10 and 50 μg/ml groups showed no significant difference compared with the control group.

When the concentrations of GO were 50, 100, 500, and 1000 μg/ml, at 0, 8, and 24 h, GO showed the effect of growth inhibition towards Xen-29. Similarly, the photon intensity in 10 and 50 μg/ml groups showed no statistically significant difference compared with the control group.

Results showed that the G presented the antibacterial ability with the concentration more than 100 μg/ml, and GO presented the antibacterial ability with the concentration more than 50 μg/ml. The antibacterial ability of G or GO was in a dose-dependent manner. GO had stronger antibacterial ability compared with G at the same concentration.

#### Antibacterial Ability In Vivo

**Fig. 6:**
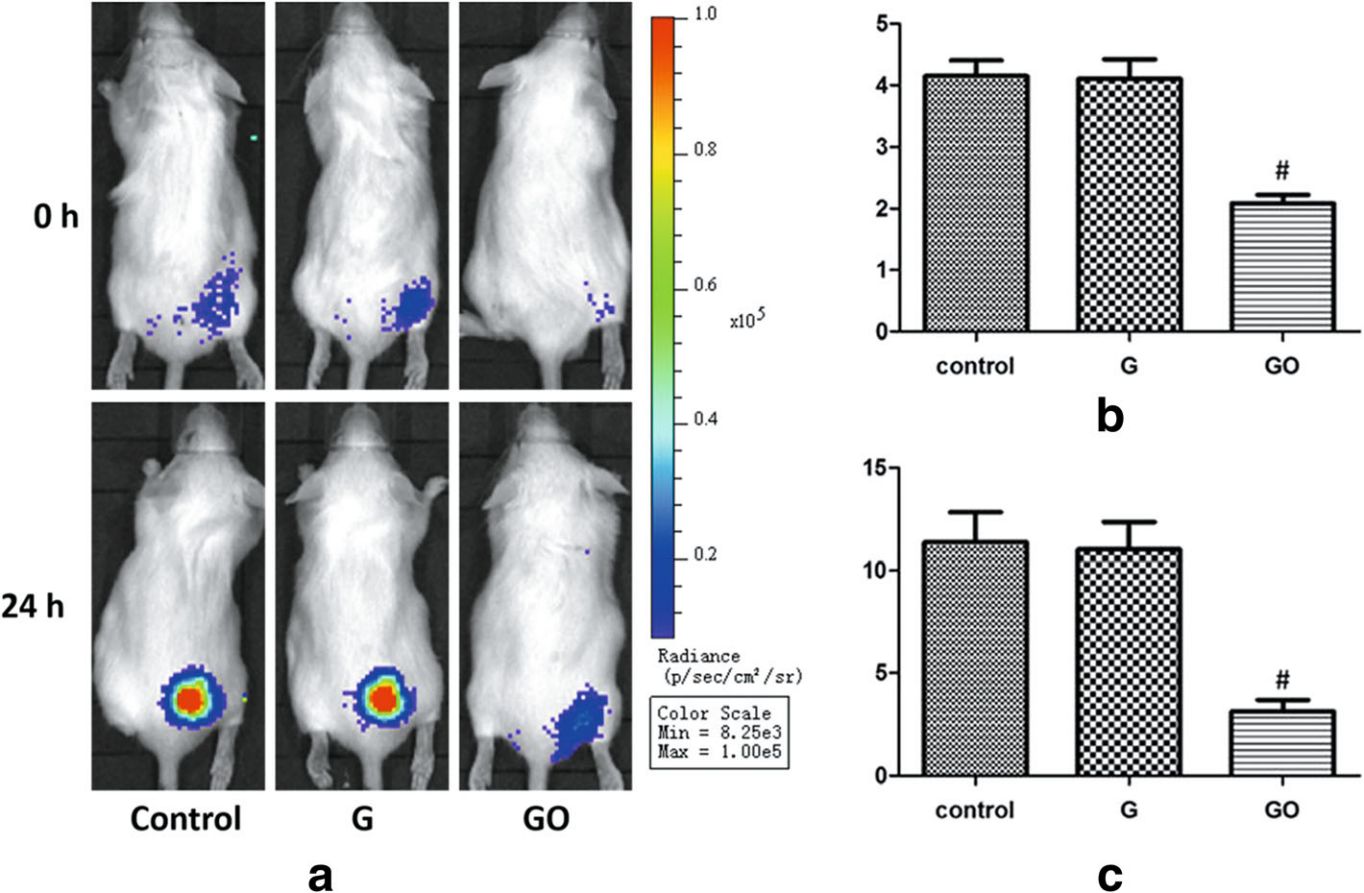
Intensity monitoring of bioluminescence of *S*. *aureus* in vivo. GO shows a dose-dependent manner in antibacterial ability in vivo. **a** Bioluminescence of Xen-29 imaged in vivo after 0 and 24 h of incubation, with variations in color representing light intensity (Bin M(8), FOV12, f1, 60 s). **b** PI = 0 h, ^#^ *P* < 0.01 with control group. **c** PI = 24 h, ^#^ *P* < 0.01 with control group

In the bacteriostasis experiment in vivo, GO group showed significantly lower photon intensity (PI) value at 0 and 24 h. The PI value was decreased compared with the G group and control group. However, PI value of G group was not statistically significantly different compared with the control group (Fig. 6). Results showed that GO showed strong antibacterial ability but G showed no obvious antibacterial ability in vivo at the 100-μg/ml concentration.

## Discussion

With the development of tissue engineering, an increasing number of biomaterial applications are used in the field of orthopedic treatment [41]. Good biosafety is necessary for biomaterials. G and GO have been used widely in the medical field for their safety and unique physical and chemical properties. On the aspect of antibacterial ability, G and GO are the good antibacterial substances. The major antibacterial mechanisms are charge transfer [28, 29] and penetration into the cells [30]. Thus, antibacterial ability of G and GO could meet the requirements for bone repair materials beneath the safe ranges.

In this study, to identify the biosafety properties, we observed G and GO cytotoxic effect towards BMSCs through SEM and TEM, and this effect presented as a dose-dependent manner. Moreover, GO had higher cytotoxic effect. On the aspect of antibacterial properties, we further observed that G and GO had antibacterial properties as a dose-dependent manner, and the GO effect was significantly better than G in vivo. In conclusion, the concentration in the range of 50~100 μg/ml may be better to keep the balance of minor cytotoxic effect and major antibacterial ability.

This research demonstrated that both G and GO present the cytotoxic effect towards BMSCs and skeletal cells and GO toxicity is higher than G. A large number of studies have demonstrated the G nano-cell toxicity and its physical and chemical properties of the material (such as size, shape, and surface functional groups) towards cells [35, 42, 43]. Besides, researchers found that pristine G can induce cytotoxicity through the depletion of the mitochondrial membrane potential (MMP) and the increase of intracellular reactive oxygen species (ROS) [12], therefore triggering apoptosis by activation of the mitochondrial pathway [34, 44]. But the phenomenon that the cytotoxicity of GO is higher than G may be associated with the groups contained on surface of GO [45]. Researchers have found that cytotoxicity of GO directly relates to serum content. Hu W et al. demonstrated that GO has strong adsorption capacity which could adsorb serum protein to form protein inclusions [46], demonstrating the higher cytotoxic basic of GO compared to G. Our experiments confirmed the conclusions mentioned above. Besides, animal toxicity is another important indicator of biological safety evaluation of G and GO. In this study, serious pathological response in muscle tissues was found in GO group, indicating its higher toxicity compared with G group.

Secondly, antibacterial properties are in line with G and GO dose changes; 50~100 μg/ml GO concentration could balance biological toxicity and antibacterial ability better. Our studies demonstrated that both biological toxicity and antibacterial ability present as the dose-dependent manner. Therefore, some concentration range may keep the balance of minor biological toxicity and major antibacterial ability.

Results showed that both G and GO had some biological toxicity towards BMSCs and muscle tissues, but in GO group, the antibacterial ability was significant in vivo. Based on the previous results of G and GO toxicity, we found that the concentration of 50~100 μg/ml may be the better one to the keep the balance of minor biological toxicity and major antibacterial ability, thus providing new evidence towards biosafety and antibacterial ability of G and GO in vivo and in vitro in clinical work.

Though GO has much toxic effect, the toxicity may be avoided by modifications of GO [47, 48]. At the same time, modified GO materials can be degraded and cleared in the body [49]; thus, a new research direction of the modification for GO is urged. Moreover, effects of G and GO towards other important organs or tissues still need further research to reach the holism medicine. Meanwhile, whether GO causes oxidative stress damage to the bacteria and the presence of additional antibacterial mechanism needs further study. Before the application to tissue engineering, mechanisms of G and GO toxicity and modified ways to reduce toxicity still need more clarification.

## Methods

### Animals

Male Sprague-Dawley (SD) rats and male Balb/C mice were purchased from Pasteur Institute of Iran and maintained under the 12-h light/dark condition at 25 °C. SD rats at the age of 4 weeks were used for isolation of the BMSCs. Balb/C mice were used for animal experiments in vivo. All the animals were raised in the Laboratory Animal Centre of Fourth Military Medical University, and operations were following the Animal Experimental Surgery Standard of Xijing Hospital. All animal experiments were approved by the Institutional Animal Care and Use Committee of the Fourth Military Medical University.

### Graphene and Graphene Oxide

G or GO (layers 1–2) (Hengqiu Graphene Technology, China) was respectively added into absolute ethanol (used for transmission electron microscope test, TEM), PBS buffer (used for in vitro cell experiments), and saline solution (used for in vivo animal experiments) to prepare the G or GO solution (Raman spectroscopy testing result was provided by Hengqiu Graphene Technology). The initial concentration of G or GO solution was 1 mg/ml. G or GO solution was dispersed using ultrasonic 2 h before the experiments.

### Cytotoxicity

#### Cell Culture

The cell culture medium contained 10% fetal bovine serum (Gibco, Carlsbad, California, USA), DMEM/F12 (Corning, NY, USA), 100 U/ml penicillin, and 100 U/ml streptomycin (Sigma, St. Louis, Missouri, USA). BMSCs were extracted from the 4-week-old male rats by the method of bone marrow culture [50]. After the execution of rat, femur and tibia were removed under aseptic condition. Medullar cavity was washed with cell culture medium; then, the mixture was centrifuged under 1500 rpm for 10 min to collect bone marrow. Bone marrow was resuspended with cell culture medium and inoculated in gelatin-coated cell culture bottles at 37 °C and 5% carbon dioxide cell incubator. Medium in cell culture bottles was changed after 48 h and non-adherent cells were removed; then, culture medium was changed once every 48 h. Third to fifth passage cells were used for next experiments.

Osteogenic differentiation and adipogenic differentiation on BMSCs were carried out with differentiation medium (Cyagen, CA, USA). After 2 weeks differentiation induction, cells were fixed with 4% formaldehyde solution for 30 min; then, alizarin red stain for osteogenic differentiation and oil red stain for adipogenic differentiation were carried out.

#### Cell Activity

BMSC suspension concentration was adjusted to 5 × l0^4^/l and cells were cultured in a 96-hole plate with 100 μl in each hole. After 24 h, the medium was replaced with cell culture medium containing G or GO with the concentrations of 0 (as control group), 10, 50, 100, 500, and 1000 μg/ml. After 24 h culture, 10 μl of alamarBlue (Bio-rad, Hercules, CA, USA) was added into each hole for further 4 h culture. Microplate (Bio-rad, Hercules, CA, USA) was used to detect the OD (optical density) values at 570 and 600 nm, and then, alamarBlue® colorimetric calculator (Bio-rad, Hercules, California, USA) was used to evaluate the rate of cell proliferation.

#### Characterization Using SEM

BMSCs were seeded in a 24-hole thin glass plate with 0.17 mm thickness and 14 mm diameter. After 24 h culture, the medium was replaced with cell culture medium containing G or GO with the concentrations of 0 (as control group), 10, 50, 100, 500, and 1000 μg/ml. Cells were continued to culture for 24 h and supernatant was removed afterwards. Cells were fixed with 2.5% glutaraldehyde solution for 24 h. Then, cells were observed by scanning electron microscope (Hitachi S-4800 SEM, JPN) after dehydration and gilding.

#### Characterization Using TEM

G and GO suspension were adjusted into 50 μg/ml. Cells were co-cultured with G or GO for 24 h and then digested by 0.25% trypsin. Supernatant was removed after centrifugation with 1000 rpm for 10 min. Cells were fixed with 2.5% glutaraldehyde solution for 24 h and slices were observed by transmission electron microscopy (FEI Tecnai G2 TEM, USA).

### Toxicity and Identification of Muscle Tissue

To analyze the G and GO cytotoxicity in vivo, we select skeletal tissue to represent and simulate local transplantation circumstance in orthopedics. G or GO was respectively injected into the medial femoral muscle tissues of Balb/C mice. Mice were killed after 7 days, and muscle tissues injected with G or GO was fixed with 10% neutral formaldehyde solution for 24 h. After alcohol dehydration, tissue was wrapped in paraffin and was sliced to perform hematoxylin and eosin (HE) staining. Slices were observed under inverted microscope (Leica DMI6000B inverted microscope, RBT).

### Antibacterial Ability

#### Bacterial Culture

We selected Xen-29 to culture for its luminescent reaction. Xen-29 was a bioluminescent bacteria of *Staphylococcus aureus* (Caliper, LS, USA) derived from ATCC-12600. Bacteria were cultured in Luria Bertani medium (LB, Sigma, St. Louis, MO, USA) containing 200 μg/ml kanamycin (Sigma, St. Louis, MO, USA) at 37 °C. A single colony was taken in LB broth at 37 °C with shaking for 2–3 h at the speed of 200 rpm. When the absorbance at 600 nm reached 0.5 (roughly equivalent to 1.44 × 108 cfu/ml) compared with the absorbance in LB broth blank, the bacteria were used for next experiment.

#### Bioluminescent Imaging

To present bioluminescent imaging, we used IVIS Lumina II cooled CCD optical macroscopic imaging system (Caliper, LS, USA). Bacterial bioluminescent signal was converted into photon intensity (PI). Living Image® 4.2 software (Caliper, LS, USA) was used for quantification of PI in regions of interest (ROI). In order to prevent movement of the mice in the imaging process, mice were anesthetized to avoid instability of the received signal.

#### Antibacterial Ability In Vitro

Xen-29 was added into the 24-hole plate and the concentration in each hole was 10^7^ cfu. Then, G or GO suspension was added to adjust the concentration into 0 (control), 10, 50, 100, 500, and 1000 μg/ml. Constant volume in each hole was 500 μl. To analyze the antibacterial ability of G and GO, the bacterial PI in the ROI was sequentially measured on 0, 8, and 24 h after the intervention.

#### Antibacterial Ability In Vivo

Based on the experiment results above, 100 μg/ml group was chosen to identify the antibacterial ability. Xen-29 suspension (200 μl) was injected into the medial femoral muscle tissues of Balb/C mice. PI of ROI was detected on 0 and 24 h after the operation.

### Data Analysis

All data were presented as the mean ± standard deviation (SD). Student’s *t* test was used for comparison between G and GO with the same concentration. One-way analysis of variance (ANOVA) was used to compare the differences between G and GO with different concentration respectively. *P* < 0.05 was considered statistically significant.

## Conclusions

In conclusion, G and GO have some biological cytotoxic effect with the dose-dependent manner. G and GO have antibacterial properties and function as the dose-dependent manner as well; the GO antibacterial properties are significantly better than G in vivo. The concentration of 50~100 μg/ml may be better to the keep the balance of minor biological toxicity and major antibacterial ability. Moreover, modifications of GO to reduce toxicity need to be clarified to contribute to G and GO applications in nanomedicine.

## Abbreviations

BMSCs: Bone marrow mesenchymal stem cells
G: Graphene
GO: Graphene oxide
HE: Hematoxylin and eosin
LB: Luria Bertani medium
NPs: Nanoparticles
PI: Photon intensity
ROS: Reactive oxygen species
SEM: Scanning electron microscope
TEM: Transmission electron microscopy
